# GRACOMICS: software for graphical comparison of multiple results with omics data

**DOI:** 10.1186/s12864-015-1461-0

**Published:** 2015-04-01

**Authors:** Minseok Seo, Joon Yoon, Taesung Park

**Affiliations:** Interdiciplinary of Bioinformatics Program, Seoul National University, 1 Gwanak-ro, Gwanak-gu, Seoul Korea; Department of Statistics, Seoul National University, 1 Gwanak-ro, Gwanak-gu, Seoul Korea

**Keywords:** GUI, Microarray, NGS, Omics, RNA-seq, SNP, Visualization

## Abstract

**Background:**

Analysis of large-scale omics data has become more and more challenging due to high dimensionality. More complex analysis methods and tools are required to handle such data. While many methods already exist, those methods often produce different results. To help users obtain more appropriate results (i.e. candidate genes), we propose a tool, GRACOMICS that compares numerous analysis results visually in a more systematic way; this enables the users to easily interpret the results more comfortably.

**Results:**

GRACOMICS has the ability to visualize multiple analysis results interactively. We developed GRACOMICS to provide instantaneous results (plots and tables), corresponding to user-defined threshold values, since there are yet no other up-to-date omics data visualization tools that provide such features. In our analysis, we successfully employed two types of omics data: transcriptomic data (microarray and RNA-seq data) and genomic data (SNP chip and NGS data).

**Conclusions:**

GRACOMICS is a graphical user interface (GUI)-based program written in Java for cross-platform computing environments, and can be applied to compare analysis results for any type of large-scale omics data. This tool can be useful for biologists to identify genes commonly found by intersected statistical methods, for further experimental validation.

**Electronic supplementary material:**

The online version of this article (doi:10.1186/s12864-015-1461-0) contains supplementary material, which is available to authorized users.

## Background

Over the last decade, success in microarray data studies has led to an expansion of large-scale omics data analyses and their data types. Vast amounts of data, in various forms, are produced for a common goal: to find genetic variants related to a phenotype of interest (e.g., disease status, etc.). In unison with technological advances, many statistical tools were developed for separate types of omics data analyses. In our study, we will illustrate the application of our tool for different omics data types.

Many microarrays studies aim to detect “gene expression signatures” specific to various human diseases by comparing expression levels between two distinct groups. The main idea is to identify overexpressed and underexpressed genes, as compared to a control group, and label them as deleterious or protective, respectively. The success of this approach in human cancer, and other diseases [1], promoted the development of many statistical methods. However, unifying the analysis results from disjointed methods cannot keep up with the explosive rate of publications concerning the specific phenotype of interest. Thus, annotation and replication studies are required in this current era. Many databases, such as the National Center for Biotechnology Information (NCBI), have been used to infer biological information from omics data and make note of novel findings that were detected as previously reported “markers.”

The popularity of another type of array-based study, focusing on single nucleotide polymorphism (SNP) association studies, has steadily increased. In fact, SNP analysis has been crucial in uncovering the genetic correlations of genomic variants with quantitative traits, complex diseases, and drug responses [2]. One well-known data source, the Wellcome Trust Case Control Consortium (WTCCC) database, which handles 14,000 cases of seven common diseases and 3,000 shared controls, has led to many influential publications. While various analysis methods have been published, and public databases such as dbSNP [3] and HapMap [4] are available, utilizing them well is another issue.

Following the footsteps of array-based approaches, an era of high-throughput sequencing began, and this technology has been applied to RNA-seq and whole exome and genome sequencing. RNA-seq has properties that are different from microarrays, for example, a high dynamic range and low background expression levels. To address these properties, several statistical methods using Poisson or negative binomial distributions have been proposed [5-7]. In the case of exome and genome sequencing, issues with missing heritability have led researchers to study more than just common variants, and various methods have now been proposed to handle rare variants [8-10].

As for visualization tools, there are only a few programs available for comparison. Multi Experiment Viewer (MeV) [11] is one of the most popular tools included in the TM4 suite, which is used to analyze microarray data. Although it supports several statistical methods of microarray data analysis, MeV provides only multiple outputs in treeview. Similar to MeV, PLINK [12] is a widely used genome association analysis toolset, but does not provide graphical interactive comparison of results.

Here, we focused on exploring the inconsistent results that can be produced from method-specific assumptions and parameters. Taking an extra step to check, understand, and interpret the different results can be challenging for scientists without computational proficiency. We aimed to ease such problems by proposing a visual comparison tool in a user-friendly environment. In addition to its accessibility, GRACOMICS can reflect a change in results according to an immediate alteration of significance levels. Such characteristics are valuable, and likely essential for effective, interactive, and integrative comparison of multiple results. Therefore, the proposed tool, GRACOMICS, provides a novel approach to visually compare several test results through graphical user interface (GUI) components.

In addition to its interactive GUI, our tool provides three distinctive layouts for comparison, including pairwise plots, summary tables, and a “heatmap-like” summary table highlighting pivotal markers, commonly detected by different methods. Two of the modules, the Pairwise Comprehensive Scatter Plots Module (Pair-CSP) and the Pairwise Detailed Scatter Plot Module (Pair-DSP), compare and contrast a pair of methods at the same time, while the third, the Multiple Results Comparison Module (Multi-RC), can handle all the employed methods (more than two) at once. Note that the user can define the top *N* significant markers (from input files) that will be used in the modules, for more interactive and efficient comparison. Furthermore, simple web-annotation functionality adds to the benefits, in terms of biological interpretation.

## Implementation

### Microarray dataset and statistical methods

For microarray studies, statistical tests were performed to detect differentially expressed genes (DEGs) between two groups: cases and controls. A pre-processing step is necessary for statistical analysis of the raw expression profiles, including background correction, global or local normalization, log-transformation, etc. Such processing steps may alter the results and should be performed only after fully understanding the platform and target probes of the analysis. We employed a microarray dataset, GSE27567 [13], from the Gene Expression Omnibus (GEO) database, consisting of 45,101 Affymetrix probes from 93 individual mice. To detect the DEGs from the microarray data, we perform two group comparison tests between tumor-bearing mice and non-transgenic controls. We employed statistical tests such as *t*-test, significant analysis of microarray (SAM) [14], permutation, and Wilcoxon rank-sum test.

### SNP dataset and statistical methods

In genome-wide association (GWA) studies, researchers focus on the positions of genetic variants that are significantly related to the phenotype of interest. There is no gold standard for pre-processing such data, but a few guidelines exist. Many steps, such as normalization and bias removal are included in data pre-processing, and the analysis results are very dependent on those steps. In our analysis, we used a bipolar disorder data in the WTCCC database, which includes 354,019 SNPs from 4,806 individuals (1,868 bipolar disorder patients and 2,938 normal controls). As a first step, we conducted a quality control process based on specific criteria [15]. For the association test between genotype and phenotype, using SNP data, we used statistical methods such as chi-square test, Fisher’s exact test, logistic regression with covariate adjusting, and logistic regression without covariate adjusting. These association tests were implemented using the PLINK tool.

### RNA-seq dataset and statistical methods

We employed results from RNA-seq, another type of transcriptome measuring platform. Recently, its advantages over microarray platforms have been described by many comparative reports [16]. Thus, a more elaborated estimation became possible by RNA-seq, in short. However, RNA-seq gene expression is measured in counts (i.e., number of strands synthesized), and therefore direct application of RNA-seq methods to microarray analysis is impossible. Instead, RNA-seq analysis methods are developed by applying statistical methodologies based on analyzing serial analysis of gene expression (SAGE) platform data, a traditional approach for measuring gene expression in counts. Here, we employed RNA-seq data from a previous study [17] using edgeR, DESeq, and NBPSeq methods. The RNA-seq data from a MicroArray Quality Control Project (MAQC) had 7 replicates and one pooled sample each from two types of samples, Ambion’s (Austin, TX, USA) human brain reference RNA, and Stratagene’s (Santa Clara, CA, USA) human universal reference RNA. After filtering out the NA values; 10,473 genes remained, with three DE-analysis methods.

### NGS dataset and statistical methods

Shortcomings of common variants in explaining the whole heritability of diseases has led to the study of rare variants. Unlike common variants, rare variant analyses, based on single genetic associations, often shows large false-negative results, unless the sample or effect sizes are very large. Hence, collapsed genotype scores for a set of rare variants are suggested for an analysis scheme. For our input, we employed the results from rare variant association tests such as C-alpha, burden test, and SKAT-O. These association tests were implemented using the FARVAT tool [18]. For illustrative purposes, we used the simulation dataset of FARVAT consisting of 100 SNPs and 16 genes which was enlarged to have 10,000 SNPs and 2,000 genes, using the same settings.

### Implementation of GRACOMICS

GRACOMICS is a java-based stand-alone program using a GUI platform. It was developed under Java because statistical analysis tools are generally developed by diverse codes such as R, SAS, etc. Java programs are renowned for their compatibility with various computing environments, are supported by all operating systems, and can easily be executed by other programs written in different computer languages. GRACOMICS can read tabular types of tab-separated values (TSV) files containing p-values for each method in columns and genetic markers in rows. Also, using simple mouse clicks, rather than command lines as input, helps bridge the gap between biology-based researchers and computer science-based researchers. Our plan was to design and implement a user-friendly program any researcher could use in any environment. The proposed tool, GRACOMICS, has the following three interactive modules with distinct features:

#### Pairwise Comprehensive Scatter Plots Module (Pair-CSP)

**Figure 1:**
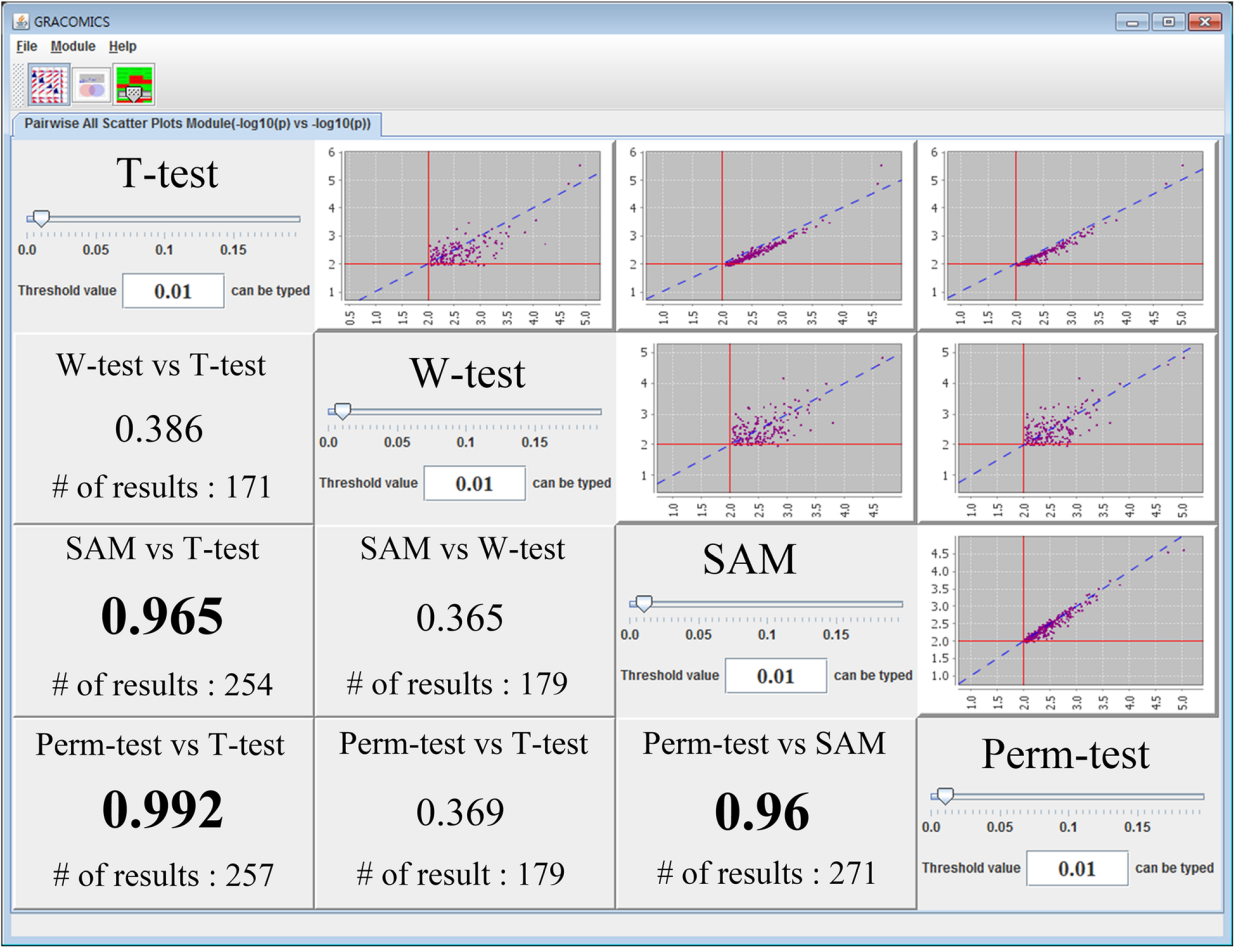
**Pair-CSP plot with GSE27567 data.** Four test results were compared, and all pairwise scatterplots and their correlation coefficients are given in the Pair-CSP module.

**Figure 2:**
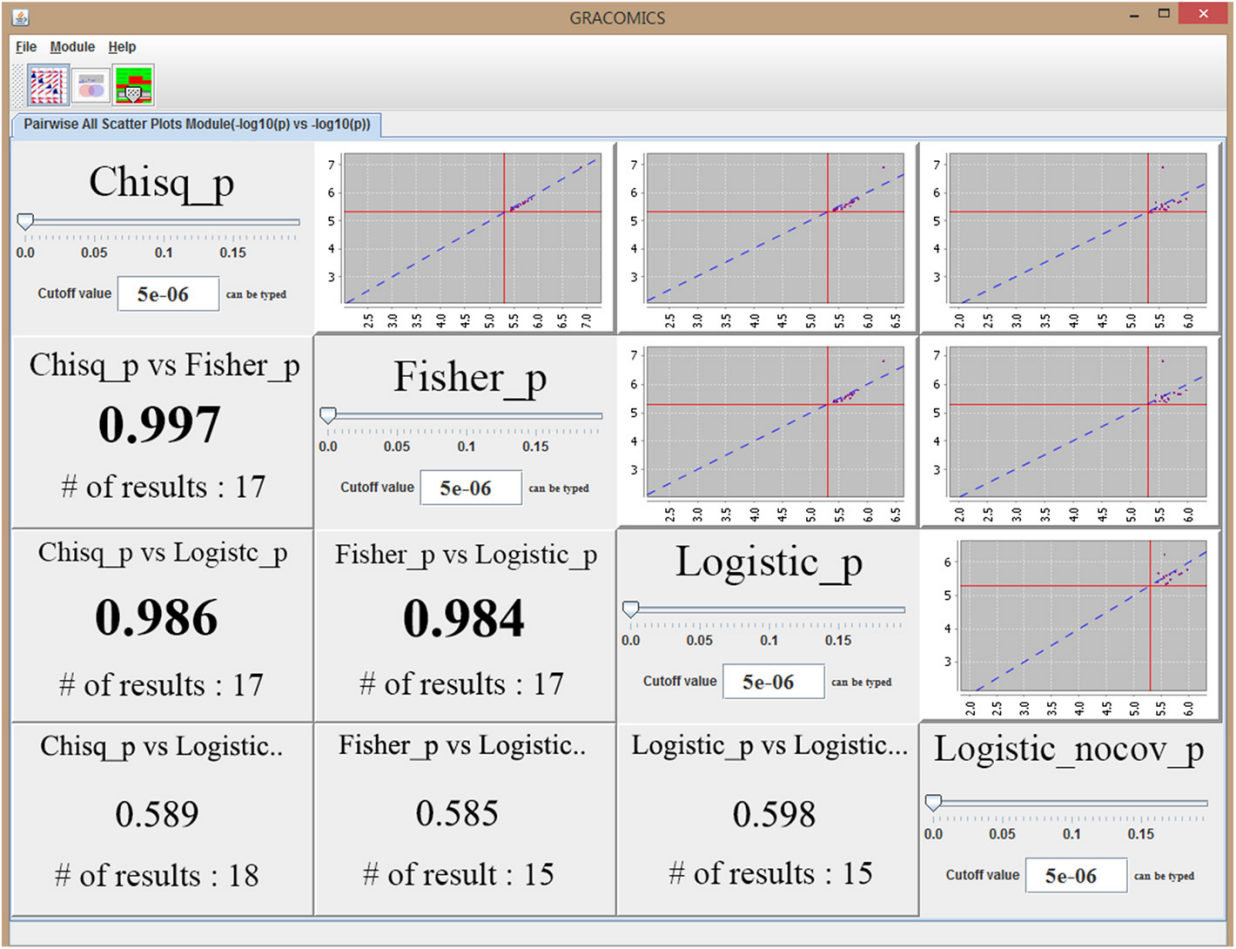
**Pair-CSP plot with WTCCC SNP data.** Four tests results were compared, and all pairwise scatterplots and their correlation coefficients are given in Pair-CSP.

Pair-CSP provides a scatter plot of pairwise comparisons between statistical method inputs simultaneously (Figures 1 and 2). Pair-CSP automatically generates these pairwise scatterplots using the p-values from the input file(s), letting the user interpret the similarities between the test results through correlation plots and correlation coefficients at a glance. When the significance level is manipulated, the pairwise scatterplots change accordingly, to display markers over the threshold only. There are two reasons behind this feature: one is to reduce computational time for drawing multitudinous points, and the other is to show only what the researcher wants to see, i.e., the meaningful results.

#### Pairwise Detailed Scatter Plot Module (Pair-DSP)

**Figure 3:**
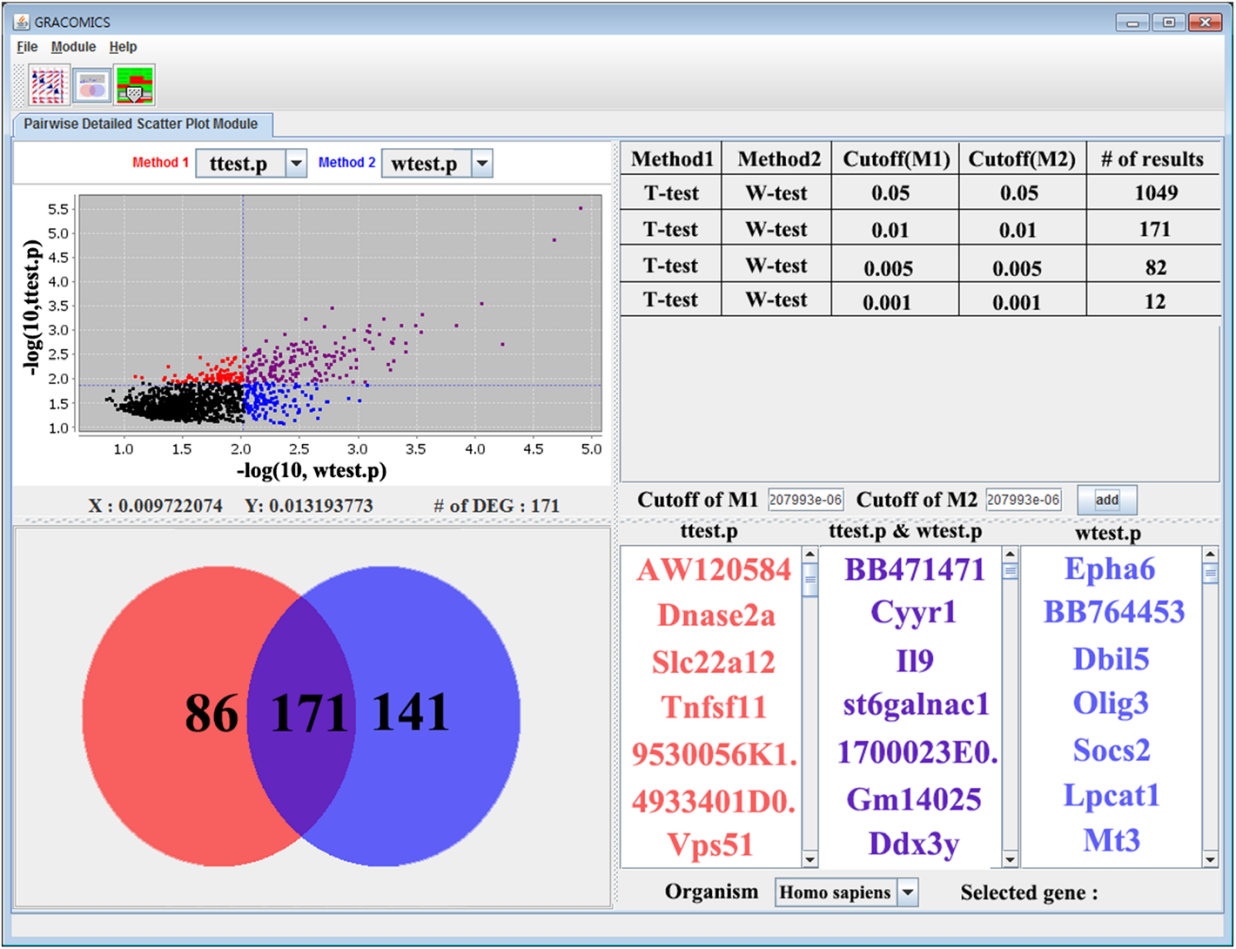
**Pair-DSP plot with GSE27567 data.** Wilcoxon rank sum tests and *t*-tests were chosen for detailed investigation, Venn diagram and the summary tables are key features of Pair-DSP.

**Figure 4:**
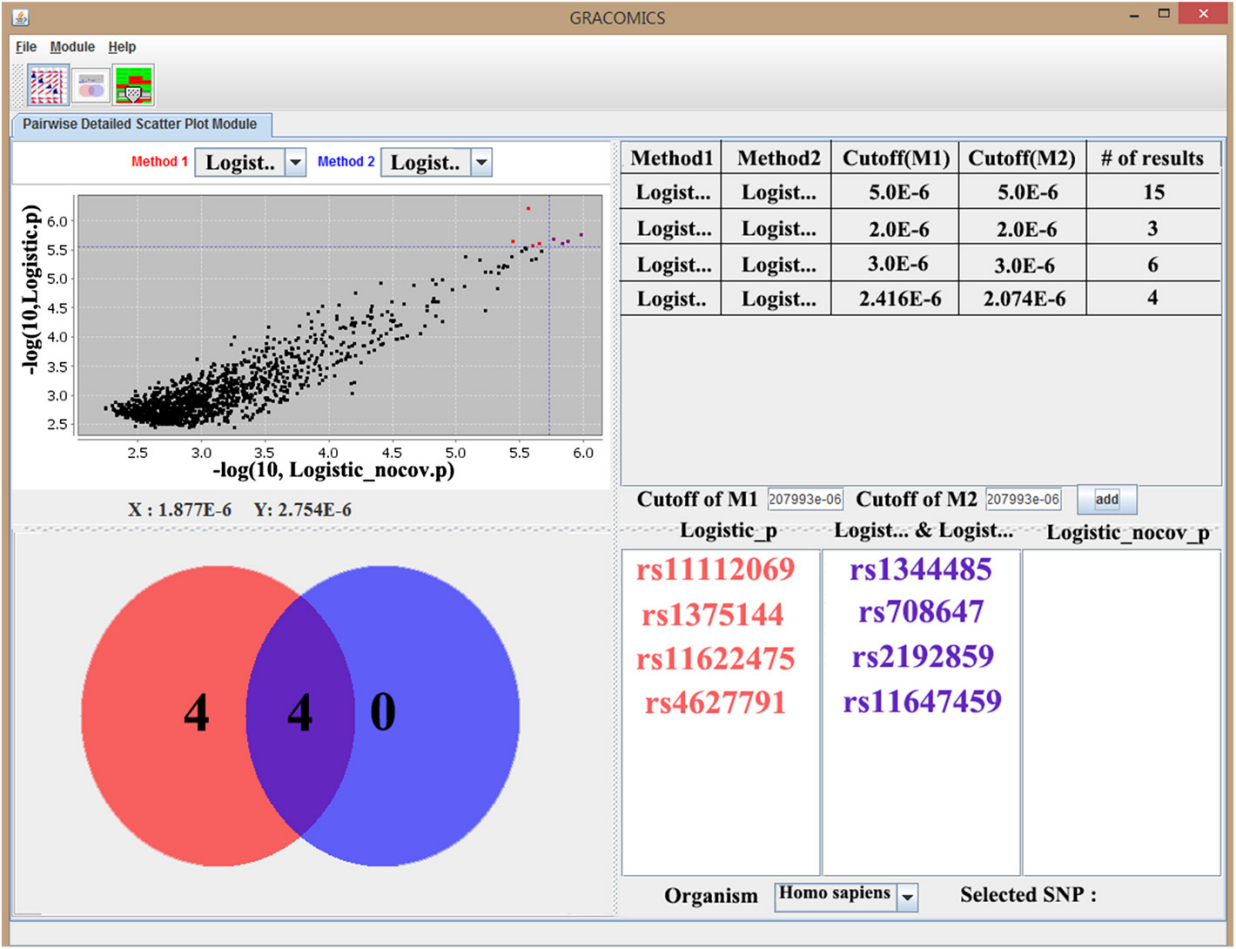
**Pair-DSP plot with WTCCC SNP data.** Two logistic models, one with and the other without covariates, has been chosen for detailed investigation, Venn diagrams and the summary tables are key features of Pair-DSP.

Pair-DSP is an interactive plot to compare the results between two methods on a more detailed level than Pair-CSP (Figures 3 and 4). This module is linked to Pair-CSP, enabling the user to directly access Pair-DSP from Pair-CSP for extended summarization of the chosen biomarkers. The summary organizes meaningful results via a Venn diagram, a table, and a marker list. For the known marker’s function, simple annotation of a single biomarker is offered via the NCBI database. Its simple annotation function automatically provides a link to the NCBI web page corresponding to its marker type, for convenience. In addition, for pathway analysis of microarray data, GRACOMICS connects to the web-based DAVID database. As a result, researchers can summarize their list of significant results, and then check the biological functions of the chosen markers.

#### Multiple Results Comparison Module (Multi-RC)

**Figure 5:**
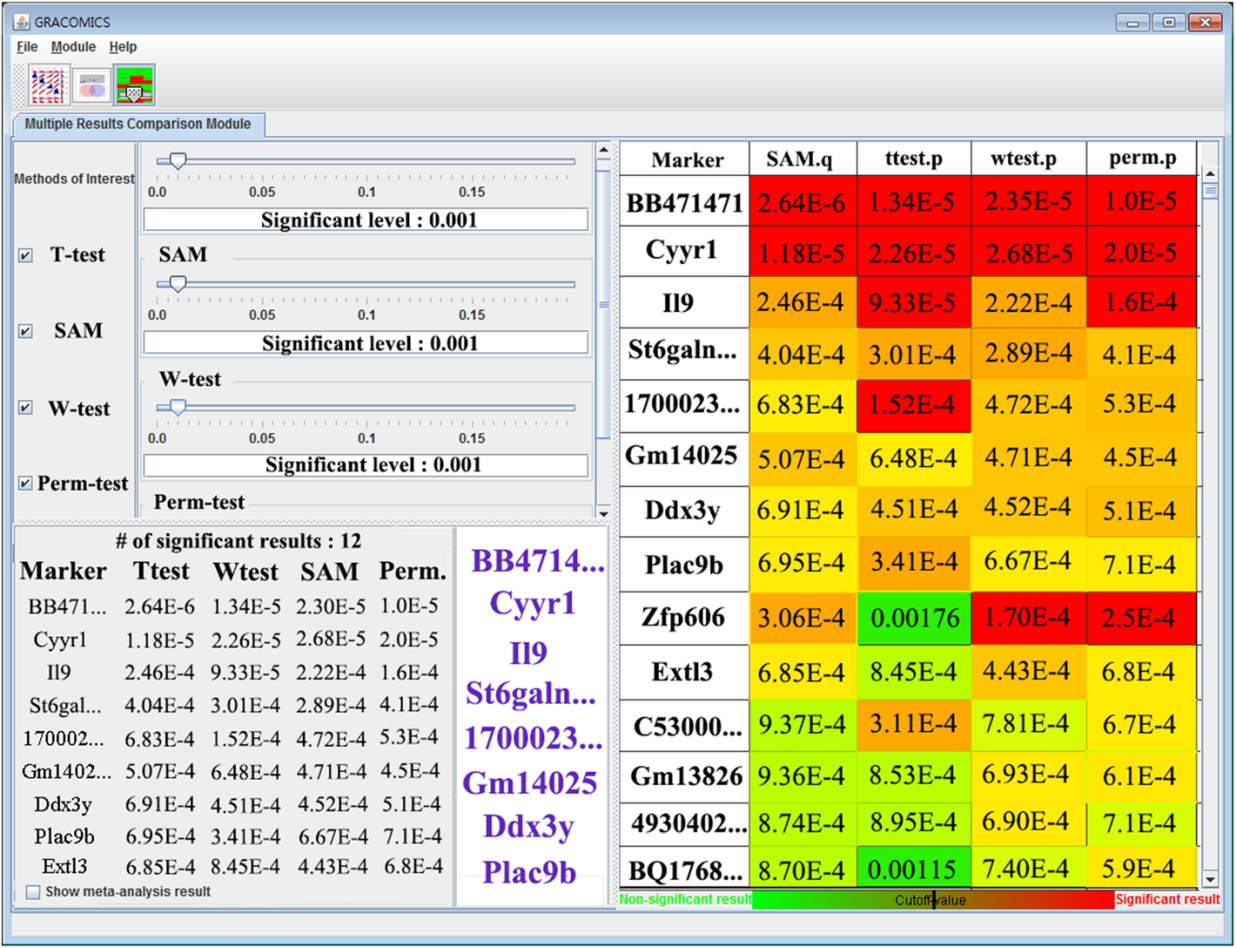
**Multi-RC plot with GSE27567 data.** The Multi-RC module provides an overall summary in a heatmap-like tabular format which highlights markers with the lowest average p-values. The user can then choose which methods to investigate by using the checkboxes in the top-left panel.

**Figure 6:**
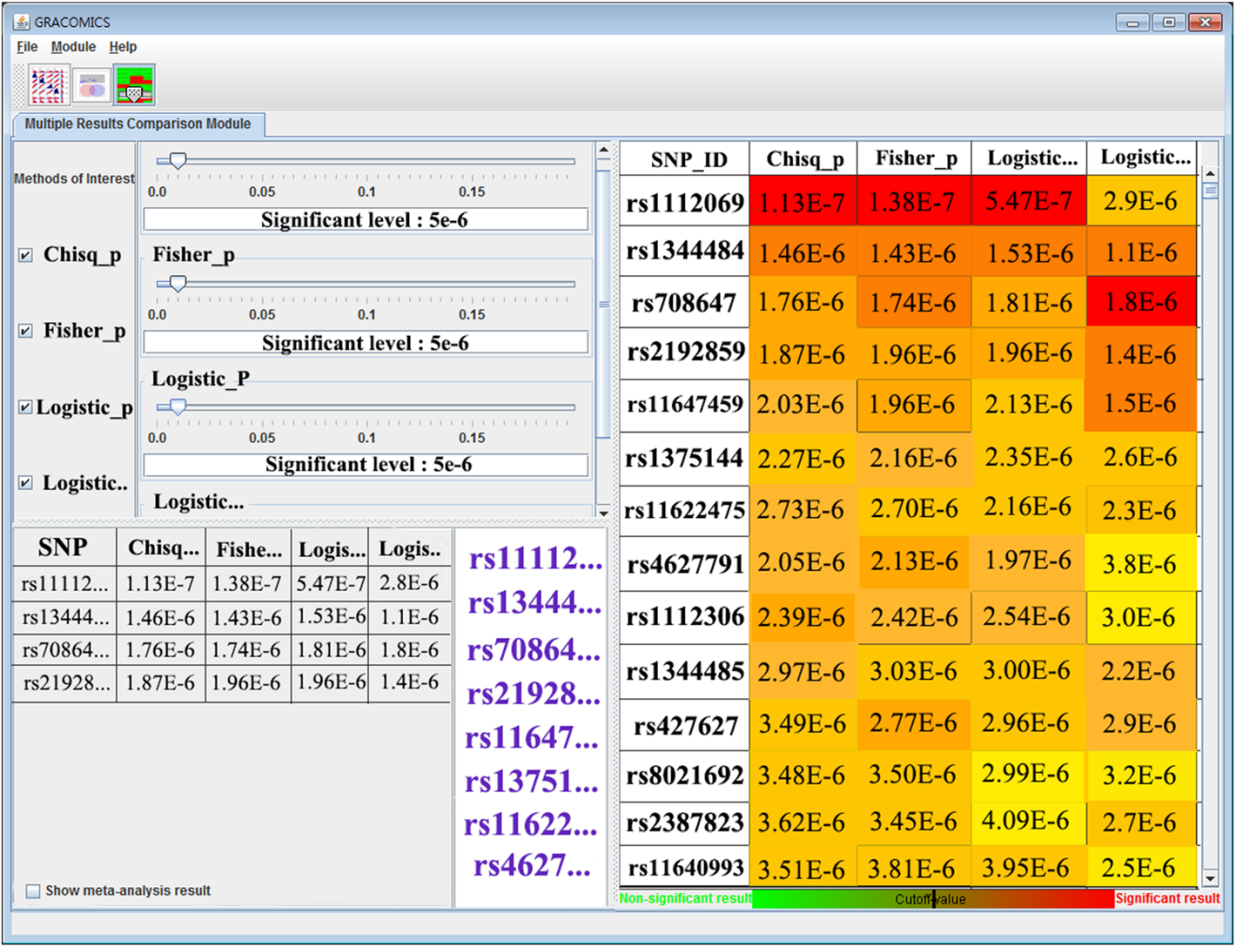
**Multi-RC plot with WTCCC SNP data.** The Multi-RC module provides an overall summary in a heatmap-like tabular format which highlights markers with the lowest average p-values. Note rs1112069 is colored in red by 3 of the 4 tests, as discussed in the manuscript.

Multi-RC provides simultaneous comparison of numerous test results (Figures 5 and 6). Researchers can choose an interesting subset of methods and set their significance levels separately. A tabular output with rows as significant markers and columns as statistical methods, is provided (with p-values in each cell). Each cell is color-coded red or green, representing significant or not, respectively. Also, variation of color intensities are used to represent the degree of significance, with more significant markers colored more intensely. In addition, Multi-RC summarizes commonly significant results and provide links to their annotation. As an extra option (with a checkbox) for meta-studies, we implemented Fisher’s method in combining p-values to provide overall importance in version 1.1.

## Results

### Application of GRACOMICS to real microarray data

In Figure 1, the plots provided by Pair-CSP compare the test results of *t*-test, Wilcoxon rank-sum test, SAM, and permutation test, displaying the top 1,500 markers by their average p-values (the user can designate the number or percentage of markers to be displayed). Pair-CSP reveals a close relationship between each pair of methods; most correlation coefficients are over 0.9, except for those with the Wilcoxon rank-sum test. Although both Wilcoxon rank-sum and permutation tests are nonparametric tests, the Wilcoxon rank-sum test uses only rank information, while the permutation test uses the variance information that arises when defining *t*-test statistics. Thus, they provide different results.

In order to compare the Wilcoxon rank-sum test to other tests more systematically, we used Pair-DSP focusing on the *t*-test and the Wilcoxon rank-sum test. As shown in Figure 3, Pair-DSP displays a pairwise plot of the two methods using p-values, and summarizes the number of genes commonly identified by the two methods. Unlike the pairwise plot of Pair-CSP, the pairwise plot of Pair-DSP shows far more detailed information. For example, a red color represents the significant genes identified by *t*-test only, a blue color signifies those identified by Wilcoxon rank-sum test only, and purple color indicates those identified by both tests. The gene name, in tool tip form, of a point is provided when the cursor is put directly over the single point. The summary table, at the top right, shows a decrease in the number of significant genes commonly identified by the two methods goes from 1,049 to 12, as the cut-off value decreases from 5% to 0.1%. Pair-DSP also provides a Venn diagram displaying the numbers of genes identified commonly and separately by the two methods. Pair-DSP shows that 171 genes remained significant by both t-tests and Wilcoxon rank-sum tests at the 1% significance level. 86 genes were significant by *t*-test only and 141 genes by Wilcoxon rank-sum test only, at the same significance level. The bottom right table shows the list of genes identified by the two methods.

To investigate the functions of the identified genes, simple annotation is provided via the NCBI database. This simple annotation function automatically opens a link to the NCBI web page corresponding to the gene of interest, for convenience. In addition, for a pathway analysis annotation database, GRACOMICS provides connection to the web-based DAVID database [19]. For example, clicking the gene *Cyyr1*, followed by a right click shows a popup window with two menus of “Link to NCBI annotation database” and “Link to DAVID annotation database”. From the NCBI database, researchers can investigate known gene functions, and related papers in PubMed, for each gene. We observed that *Cyyr1* [20] and *Il9* [21] are genes reported in PubMed. Next, when using DAVID to analyze the functional annotation of the 171 commonly identified genes from t-tests and Wilcoxon rank-sum tests, we observed the gene list to be enriched in the GO term “cell cycle arrest,” with a p-value of 4.1e-3. As a result, researchers can summarize their list of significant results, and then check the biological functions and related publications of the chosen markers.

The Multi-RC module allows simultaneous comparison of two or more results, as shown in Figure 5. We selected four methods: *t*-test, SAM, Wilcoxon rank-sum test, and permutation test, with a cut-off value of 0.1%. In this setting, we observed 12 common significant genes between all the methods. The genes *BB471471*, *Cyyr1*, *Il9*, and *St6galnac1* [22] were consistent candidates from all four methods. However, while *BB471471* was at the top of the list, no reports were found of its association with tumours or any other diseases. Therefore, we suggest the *BB471471* is a worthy candidate to examine further for its possible association with tumours. By analyzing this real microarray data analysis with GRACOMICS, we identified several commonly significant DEGs from comparisons from each method, to obtain the most reliable candidate DEGs.

### Application of GRACOMICS to real SNP data

In Figure 2, the plots are provided by Pair-CSP, which compares the test results of chi-square test, Fisher’s exact test, and logistic regression analyses. In the figure, two results from logistic regression analyses are provided: one is without covariates and the other is with the adjusting covariate effects of sex, age and the first two principal components. Although the significance of covariates can be easily tested, it is not always straightforward to determine which adjusting covariates to include in the model [23]. Here, we focused on the results from the two logistic models and demonstrate how efficiently GRACOMICS can be used to compare these two results, showing that the correlation between the two logistic regression models was 0.598.

For a further detailed comparison between these two results, Pair-DSP, in Figure 4, was conducted on these two logistic models. The summary table, at the top right, shows that the number of significant genes commonly identified by the two methods gradually decreases from 15 to 4, as the cut-off value decreases from 5.0e-6 to 2.4e-6. The Venn diagram illustrates that Pair-DSP successfully identified rs1344484 [24], rs708647, rs2192859 [25], rs11647459 and rs4627791 [26], in purple, as the most commonly detected SNPs. The four SNPs in red, rs11112069, rs1375144, rs11622475, and rs4627791, were detected by the with-covariates model only. We found rs11112069 as the top result (in average p-value), with low p-values in all four analyses. This SNP is within intron-2 of *CHST11*, a gene which has previously been reported as bipolar disorder-associated [27].

In the next module (Multi-RC; Figure 6), users can see the change in p-values for each marker, according to the method used or adjustments for covariates. Rs11112069 is displayed at the top of the list, and is marked in red (very significant) from 3 of the 4 tests, with a fairly low p-value for the fourth test also. To further analyze the top results, GRACOMICS can automatically distinguish marker types and links to dbSNP in the NCBI database for selected SNPs. From the annotation, researchers can attain detailed SNP information, such as location of the SNP, its mapped gene, clinical significance, etc. Unlike the microarray example, DAVID is not directly applicable to SNP data. However, we expect that other annotation databases will be added to future updates.

### Application of GRACOMICS to real RNA-seq data

As shown in Additional file 1: Figure S1, Pair-CSP shows that all three analysis methods; edgeR, DESeq, and NBPSeq, yield very similar results. All of the correlation coefficients are over 0.86, and the highest was between edgeR and DESeq. In addition, the plots illustrate that edgeR generates lower P-values than the others, due to scattered points being skewed toward the y-axis (edgeR). Under the 1% significance level, approximately 7,000 genes were detected as DEGs by each method. In Additional file 2: Figure S2, Pair-DSP shows that more DEGs were identified by edgeR, as compared to DESeq. In the Venn diagram, significant genes that intersected ranged from 7087 to 1621, when decreasing the cut-off values from 0.01 to 1.0E-100. Finally, we can observe that most of genes are very significant in Multi-RC. As shown in Additional file 3: Figure S3, 6983 genes were detected by all the methods under a 1% significant level. Here, the gene symbol of RNA-seq data is its Ensemble ID, and these should be converted to official gene symbols for successful functional annotation. Although implementing the Ensemble annotation function on the web is possible, we did not include it in the current version of GRACOMICS, because accommodating several symbols in the program may lead to user confusion. Although we determined that only official gene symbols should be accommodated in the tool, later versions can be updated with such functions, if there are user demands.

### Application of GRACOMICS to simulated NGS data

Using a simulated rare variant dataset, we successfully cataloged significant genes that were test-specific or marginal in all tests. The results are shown in Additional file 4: Figures S4, Additional file 5: Figure S5 and Additional file 6: Figure S6. In this analysis, gene names were masked as Genes 1 ~ 2000 and therefore could not be annotated to NCBI or DAVID. However, if a real dataset is used, the genes can be annotated in similar fashion as microarray and RNA-seq datasets. In accord with the above three applications, we could infer which methods showed higher correlation, in terms of p-values, from the Pair-CSP, followed by a detailed comparison of the number of significant genes detected in each method, and finally, by comparing the p-values in a tabular heatmap form. Here, we observed the highest correlation of 0.961 between the C-Alpha and SKAT-O methods, and these two methods shared 129 genes with a p-value threshold of < 0.05. The top-ranked genes all showed p-values ~ 0.001 using all the methods, and would be candidates of interest for end-users if this was a real data analysis.

## Discussion

From the aforesaid illustration, we demonstrated the potential of GRACOMICS to successfully highlight biologically meaningful results from multiple methods. Traditional bioinformatics studies, and some recent works, show that simple comparison of results has been widely used for biological interpretation. For example, a transcriptome study concluded that in a situation where the most reliable list of markers is desirable, the best approach was to examine the intersection of genes identified by all tried methods, or by more conservative tests. Since checking the underlying assumptions of all methods is not easy, and even if the assumptions are met, each method may provide different results, which are hard to interpret. The easiest and most conventional method is to find commonly identified markers to trim down the candidate list, and carry on further analysis. While GRACOMICS cannot give conclusive evidence that the highlighted markers are significant, it can help the biologist narrow down the candidate list, based on the intersection of markers for efficiency for further validations, such as RT-PCR.

In addition to comparison of multiple results of the same datasets, GRACOMICS can be applicable to other types of studies. First, GRACOMICS can compare the results from different datasets, such as different tissues or organs. An RNA study compared differentially expressed test results from various tissues, such as liver, adipose tissue, muscle, and brain. GRACOMICS can effectively provide the list of common genes, as well as tissue-specific genes. Second, GRACOMICS can compare results from different platforms, such as microarray vs. RNA-seq [28]. Here, GRACOMICS can trim down the list of candidates significant to both platform results, for further biological validation. Finally, meta-analysis combining independent results from different studies can be analyzed by GRACOMICS; the p-values from each study can be efficiently compared to others and can be combined easily by Fisher’s method. For meta-analysis, the compared results should be from independent datasets. However, when one single dataset was analyzed by multiple methods, the independent assumption is violated; the interpretation of this Fisher’s combined p-value should be made with caution.

## Conclusions

Comparative study of omics data analyses is unavoidable; however, many researchers skip the comparative step because it is a complicated process. GRACOMICS enables easy comparison of several methods for analyzing specific omics data platforms by any user. The four omics data employed are active areas of study in bioinformatics. We employed microarray & RNA-seq data at the transcriptomic level, and SNP and NGS data at the genomic level, to display the utility of GRACOMICS. So far, GRACOMICS can also employ proteomic analysis results, and will be extended to accommodate other types of annotations for proteomics data in a future study. In summary, we believe that this will be a highly valuable and straightforward tool for non-computational biologists, strongly assisting them in their interpretation of results from new cutting-edge technologies.

### Availability and requirements

**Project name:** GRACOMICS

**Project home page:**http://bibs.snu.ac.kr/software/GRACOMICS

**Operating system:** Platform-independent

**Programming language:** Java

**Other requirements:** Java 1.7.0_45 or higher

**License:** LGPL 2.1

## Additional files

Additional file 1: Figure S1. Pair-CSP plot with MAQC RNA-seq data. Three tests results have been compared, and all pairwise scatterplots and their correlation coefficients are given on Pair-CSP. Additional file 2: Figure S2. Pair-DSP plot with MAQC RNA-seq data. EdgeR and DESeq were chosen for detailed investigation. Venn diagrams and the summary tables are key features of Pair-DSP. Additional file 3: Figure S3. Multi-RC plot with MAQC RNA-seq data. The Multi-RC module provides an overall summary in a heatmap-like tabular format which highlights markers with the lowest average p-values. Additional file 4: Figure S4. Pair-CSP plot with simulated NGS data. Three tests results were compared, and all pairwise scatterplots and their correlation coefficients are given on the Pair-CSP GUI. Additional file 5: Figure S5. Pair-DSP plot with simulated NGS data. C-alpha and SKAT-O were chosen for detailed investigation. Venn diagrams and the summary tables are key features of Pair-DSP. Additional file 6: Figure S6. The Multi-RC plot with simulated NGS data. The Multi-RC module provides an overall summary in a heatmap-like tabular format which highlights markers with the lowest average p-values.

## Abbreviations

SNP: Single nucleotide polymorphism
GUI: Graphic user interface
NCBI: National center for biotechnology information
WTCCC: Wellcome trust case control consortium
Mev: Multi experiment viewer
Pair-CSP: Pairwise comprehensive scatter plots module
Pair-DSP: Pairwise detailed scatter plot module
Multi-RC: Multiple results comparison module
DEGs: Differentially expressed genes
GEO: Gene expression omnibus
SAM: Significant analysis of microarray
GWA: Genome-wide association
TSV: Tab separated values TSV
SAGE: Serial analysis of gene expression
MAQC: Microarray quaility control project

## References

[1] Pan W (2002). A comparative review of statistical methods for discovering differentially expressed genes in replicated microarray experiments. Bioinformatics.

[2] Hirschhorn JN, Daly MJ (2005). Genome-wide association studies for common diseases and complex traits. Nature Reviews Genetics.

[3] Sherry ST, Ward M-H, Kholodov M, Baker J, Phan L, Smigielski EM, Sirotkin K (2001). dbSNP: the NCBI database of genetic variation. Nucleic acids research.

[4] Gibbs RA, Belmont JW, Hardenbol P, Willis TD, Yu F, Yang H, Ch'ang L-Y, Huang W, Liu B, Shen Y (2003). The international HapMap project. Nature.

[5] Anders S, Huber W (2010). Differential expression analysis for sequence count data. Genome biol.

[6] Di Y, Schafer DW, Cumbie JS, Chang JH (2011). The NBP negative binomial model for assessing differential gene expression from RNA-Seq. Statistical Applications in Genetics and Molecular Biology.

[7] Robinson MD, McCarthy DJ, Smyth GK (2010). edgeR: a Bioconductor package for differential expression analysis of digital gene expression data. Bioinformatics.

[8] Neale BM, Rivas MA, Voight BF, Altshuler D, Devlin B, Orho-Melander M, Kathiresan S, Purcell SM, Roeder K, Daly MJ (2011). Testing for an unusual distribution of rare variants. PLoS genetics.

[9] Wu MC, Lee S, Cai T, Li Y, Boehnke M, Lin X (2011). Rare-variant association testing for sequencing data with the sequence kernel association test. The American Journal of Human Genetics.

[10] Lee S, Emond MJ, Bamshad MJ, Barnes KC, Rieder MJ, Nickerson DA, Team ELP, Christiani DC, Wurfel MM, Lin X (2012). Optimal unified approach for rare-variant association testing with application to small-sample case-control whole-exome sequencing studies. The American Journal of Human Genetics.

[11] Howe E, Holton K, Nair S, Schlauch D, Sinha R, Quackenbush J (2010). Mev: multiexperiment viewer.

[12] Purcell S, Neale B, Todd-Brown K, Thomas L, Ferreira MA, Bender D, Maller J, Sklar P, De Bakker PI, Daly MJ (2007). PLINK: a tool set for whole-genome association and population-based linkage analyses. The American Journal of Human Genetics.

[13] LaBreche HG, Nevins JR, Huang E (2011). Integrating factor analysis and a transgenic mouse model to reveal a peripheral blood predictor of breast tumors. BMC medical genomics.

[14] Tusher VG, Tibshirani R, Chu G (2001). Significance analysis of microarrays applied to the ionizing radiation response. Proceedings of the National Academy of Sciences.

[15] Oh S, Lee J, Kwon M-S, Weir B, Ha K, Park T (2012). A novel method to identify high order gene-gene interactions in genome-wide association studies: Gene-based MDR. BMC bioinformatics.

[16] Morozova O, Hirst M, Marra MA (2009). Applications of new sequencing technologies for transcriptome analysis. Annual review of genomics and human genetics.

[17] Bullard JH, Purdom E, Hansen KD, Dudoit S (2010). Evaluation of statistical methods for normalization and differential expression in mRNA-Seq experiments. BMC bioinformatics.

[18] Choi S, Lee S, Nöthen MM, Lange C, Park T, Won S (2014). FARVAT: a family-based rare variant association test. Bioinformatics.

[19] Huang DW, Sherman BT, Lempicki RA (2008). Systematic and integrative analysis of large gene lists using DAVID bioinformatics resources. Nature protocols.

[20] Vitale L, Frabetti F, Huntsman SA, Canaider S, Casadei R, Lenzi L, Facchin F, Carinci P, Zannotti M, Coppola D (2007). Sequence. BMC cancer.

[21] Nagato T, Kobayashi H, Kishibe K, Takahara M, Ogino T, Ishii H, Oikawa K, Aoki N, Sato K, Kimura S (2005). Expression of interleukin-9 in nasal natural killer/T-cell lymphoma cell lines and patients. Clinical cancer research.

[22] PATANI N, Jiang W, MOKBEL K (2008). Prognostic utility of glycosyltransferase expression in breast cancer. Cancer Genomics-Proteomics.

[23] Troyanskaya OG, Garber ME, Brown PO, Botstein D, Altman RB (2002). Nonparametric methods for identifying differentially expressed genes in microarray data. Bioinformatics.

[24] Palo OM (2010). Genetic background of bipolar disorder and related cognitive impairments.

[25] Kwon M-S, Park M, Park T (2014). IGENT: efficient entropy based algorithm for genome-wide gene-gene interaction analysis. BMC medical genomics.

[26] Jiang Y, Zhang H (2011). Propensity score‐based nonparametric test revealing genetic variants underlying bipolar disorder. Genetic epidemiology.

[27] Chen Y-H, Lu R-B, Hung H, Kuo P-H (2014). Identifying Potential Regions of Copy Number Variation for Bipolar Disorder. Microarrays.

[28] Nookaew I, Papini M, Pornputtpong N, Scalcinati G, Fagerberg L, Uhlén M, Nielsen J (2012). A comprehensive comparison of RNA-Seq-based transcriptome analysis from reads to differential gene expression and cross-comparison with microarrays: a case study in Saccharomyces cerevisiae. Nucleic acids research.

