# Supplementary figures and images for: GRACOMICS: software for graphical comparison of multiple results with omics data
Source: BMC Genomics. 2015 Apr 1;16(1):256. doi: 10.1186/s12864-015-1461-0 (PMC4387734; doi:10.1186/s12864-015-1461-0)

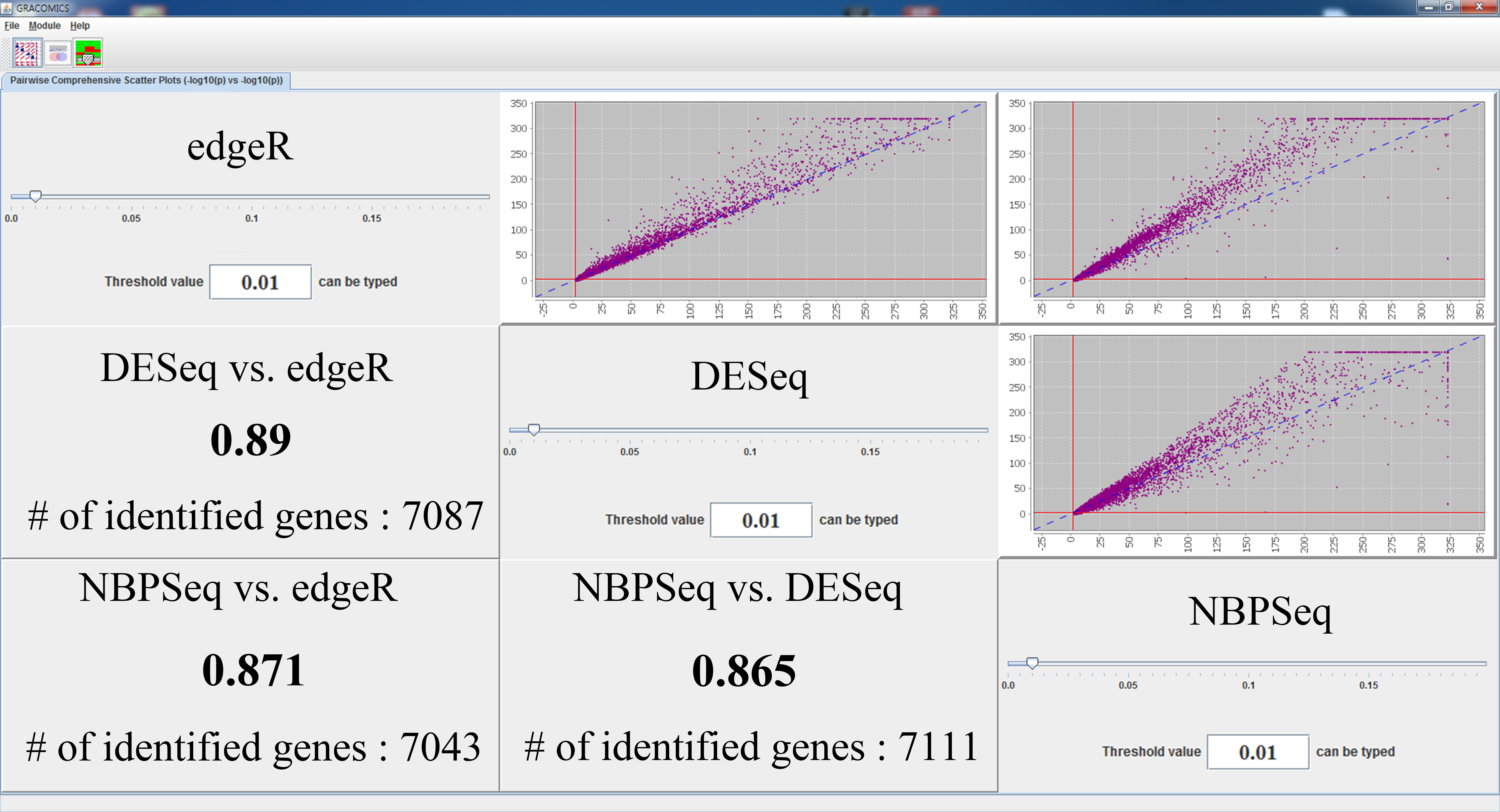

Supplement: Additional file 1: Figure S1. — Pair-CSP plot with MAQC RNA-seq data. Three tests results have been compared, and all pairwise scatterplots and their correlation coefficients are given on Pair-CSP. [file 12864_2015_1461_MOESM1_ESM.tiff]

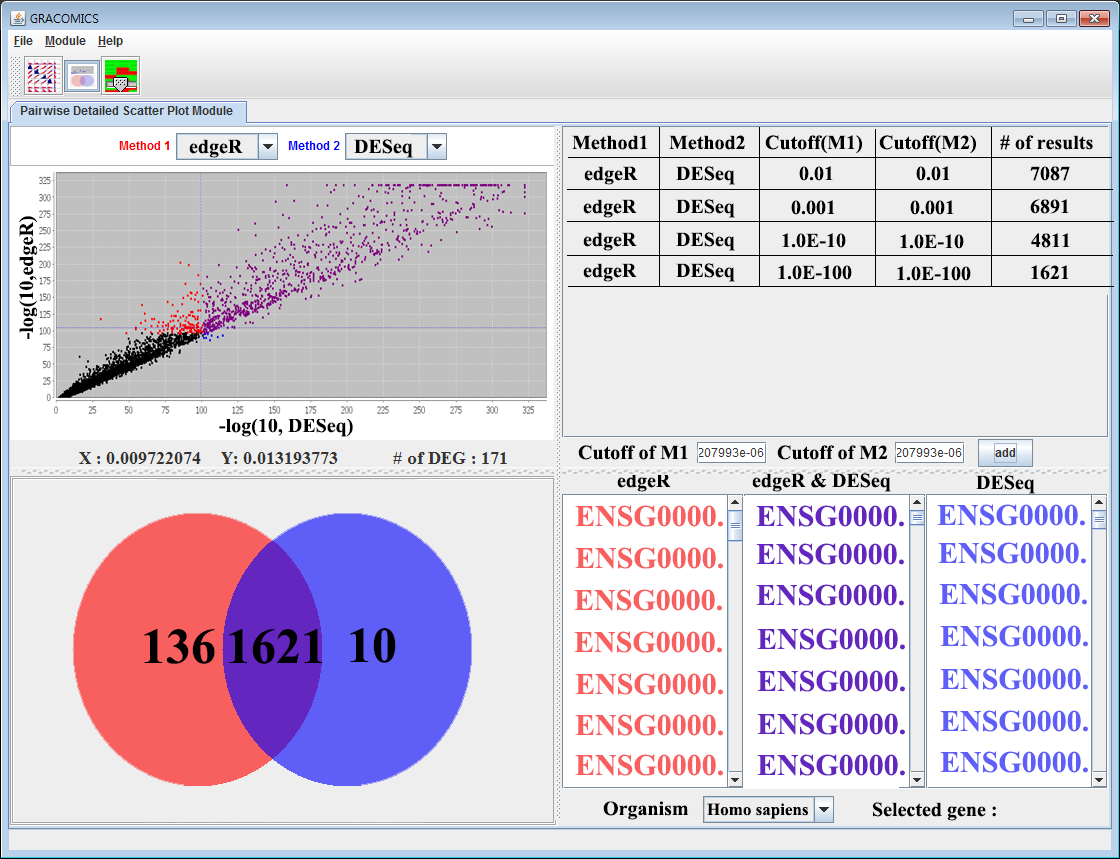

Supplement: Additional file 2: Figure S2. — Pair-DSP plot with MAQC RNA-seq data. EdgeR and DESeq were chosen for detailed investigation. Venn diagrams and the summary tables are key features of Pair-DSP. [file 12864_2015_1461_MOESM2_ESM.tiff]

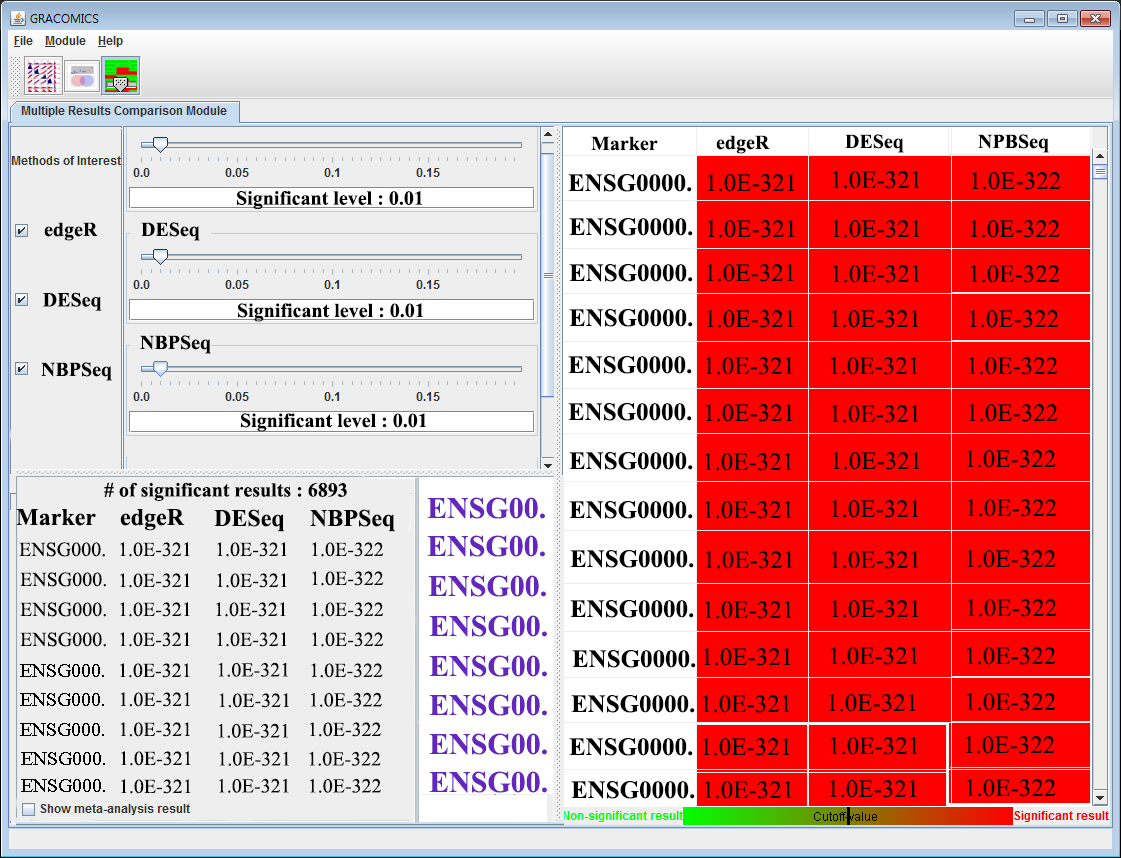

Supplement: Additional file 3: Figure S3. — Multi-RC plot with MAQC RNA-seq data. The Multi-RC module provides an overall summary in a heatmap-like tabular format which highlights markers with the lowest average p-values. [file 12864_2015_1461_MOESM3_ESM.tiff]

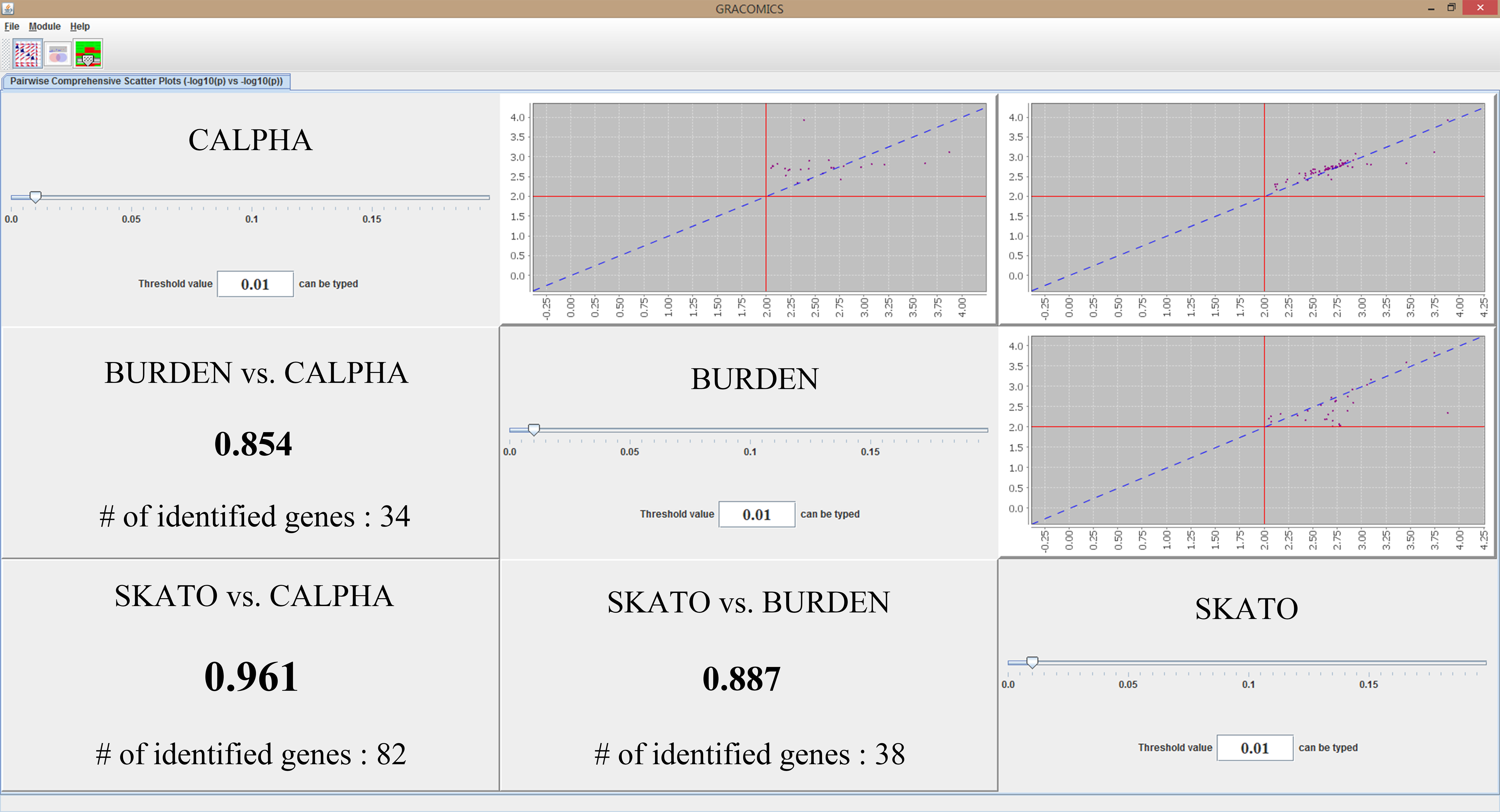

Supplement: Additional file 4: Figure S4. — Pair-CSP plot with simulated NGS data. Three tests results were compared, and all pairwise scatterplots and their correlation coefficients are given on the Pair-CSP GUI. [file 12864_2015_1461_MOESM4_ESM.tiff]

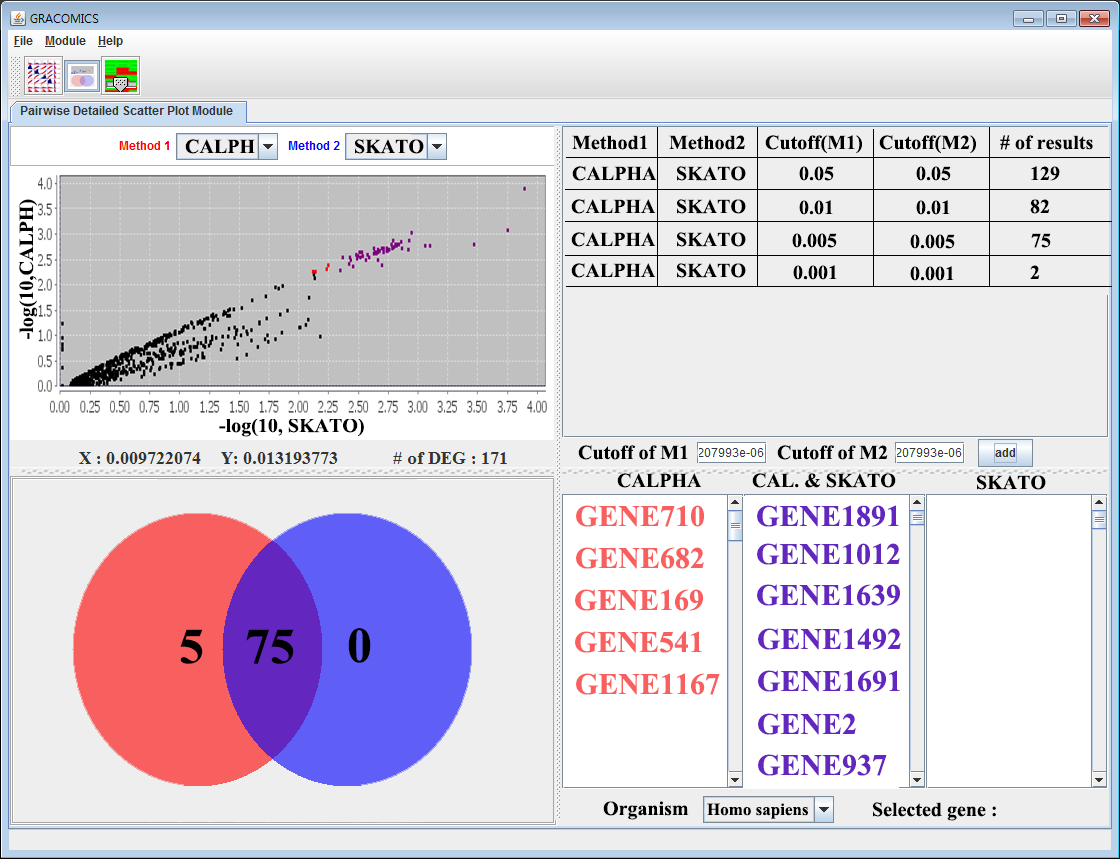

Supplement: Additional file 5: Figure S5. — Pair-DSP plot with simulated NGS data. C-alpha and SKAT-O were chosen for detailed investigation. Venn diagrams and the summary tables are key features of Pair-DSP. [file 12864_2015_1461_MOESM5_ESM.tiff]

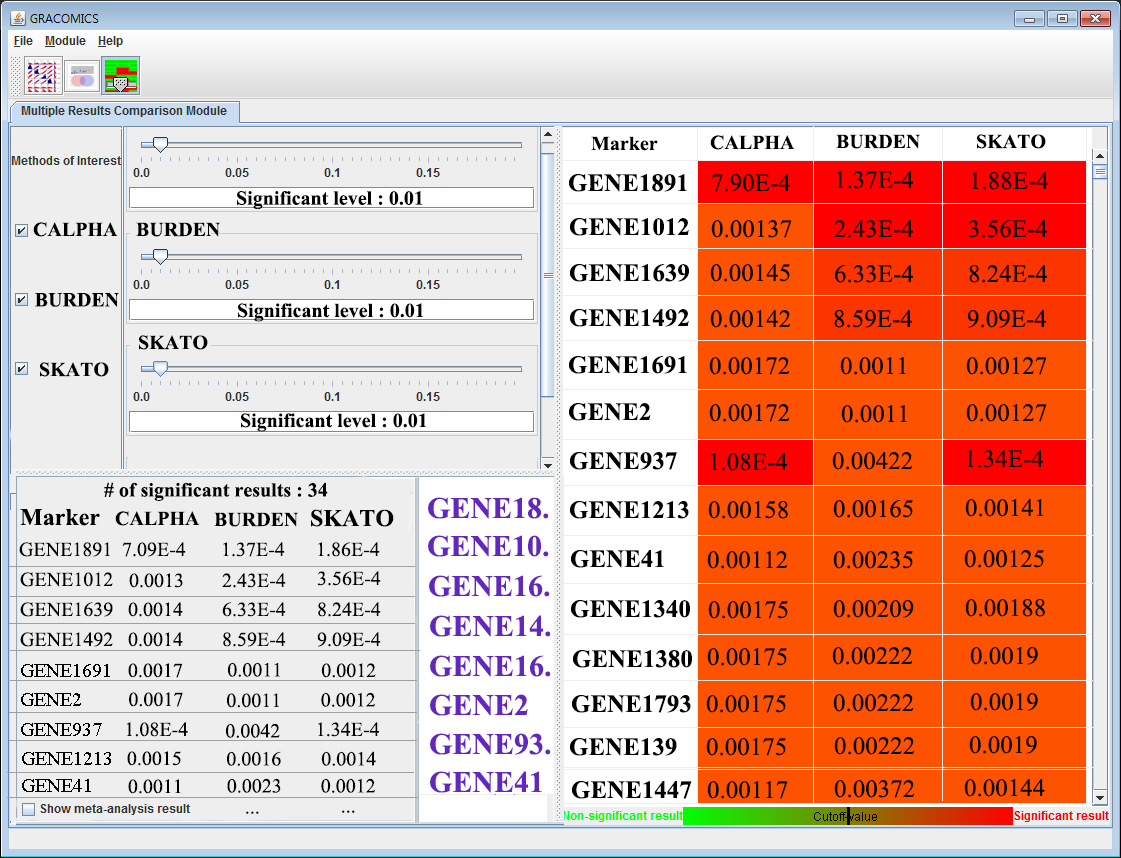

Supplement: Additional file 6: Figure S6. — The Multi-RC plot with simulated NGS data. The Multi-RC module provides an overall summary in a heatmap-like tabular format which highlights markers with the lowest average p-values. [file 12864_2015_1461_MOESM6_ESM.tiff]
